# Loss of 4q21.23-22.1 Is a Prognostic Marker for Disease Free and Overall Survival in Non-Small Cell Lung Cancer

**DOI:** 10.1371/journal.pone.0113315

**Published:** 2014-12-11

**Authors:** Faik G. Uzunoglu, Ebba Dethlefsen, Annkathrin Hanssen, Michaela Wrage, Lena Deutsch, Katharina Harms-Effenberger, Yogesh K. Vashist, Matthias Reeh, Guido Sauter, Ronald Simon, Maximillian Bockhorn, Klaus Pantel, Jakob R. Izbicki, Harriet Wikman

**Affiliations:** 1 Department of General, Visceral and Thoracic Surgery, University Medical Center of Hamburg-Eppendorf, Hamburg, Germany; 2 Institute of Tumour Biology, University Medical Center of Hamburg-Eppendorf, Hamburg, Germany; 3 Department of Pathology, University Medical Center of Hamburg-Eppendorf, Hamburg, Germany; Roswell Park Cancer Institute, United States of America

## Abstract

This study was performed to assess the prognostic relevance of genomic aberrations at chromosome 4q in NSCLC patients. We have previously identified copy number changes at 4q12-q32 to be significantly associated with the early hematogenous dissemination of non-small cell lung cancer (NSCLC), and now aim to narrow down potential hot-spots within this 107 Mb spanning region. Using eight microsatellite markers at position 4q12-35, allelic imbalance (AI) analyses were performed on a preliminary study cohort (*n = *86). Positions indicating clinicopathological and prognostic associations in AI analyses were further validated in a larger study cohort using fluorescence *in situ* hybridization (FISH) in 209 NSCLC patients. Losses at positions 4q21.23 and 4q22.1 were shown to be associated with advanced clinicopathological characteristics as well as with shortened disease free (DFS) and overall survival (OS) (DFS: *P = *0.019; OS: *P = *0.002). Multivariate analyses identified the losses of 4q21.23-22.1 to be an independent prognostic marker for both DFS and OS in NSCLC (HR 1.64–2.20, all *P<*0.04), and especially in squamous cell lung cancer (*P<*0.05). A case report study of a lung cancer patient further revealed a loss of 4q21.23 in disseminated tumor cells (DTCs). Neither gains at the latter positions, nor genomic aberrations at 4q12, 4q31.2 and 4q35.1, indicated a prognostic relevance. In conclusion, our data indicate that loss at 4q21.23-22.1 in NSCLC is of prognostic relevance in NSCLC patients and thus, includes potential new tumor suppressor genes with clinical relevance.

## Introduction

Genomic instability is one of the key hallmarks of cancer [1]. Numerous studies on genomic instability in non-small cell lung cancer (NSCLC) have highlighted specific deletion patterns for both metastatic and primary tumors [2]–[4]. Copy number aberrations in NSCLC can be found at multiple regions, some being specific for the histological subtype, and some being dependent on the severity of histopathological changes (e.g. tumor stage or grading) [5]–[11] Furthermore, the occurrence of copy number changes has shown to be an early event in lung cancer pathogenesis. This can be found in combination with a sequential pattern of loss of heterozygosity (LOH), which begins with the allelic loss on chromosome 8p, followed by 3p and 9p deletions [7], [12], [13].

We have previously shown that early hematogenous dissemination of tumor cells is driven by a specific pattern of genomic changes. In addition to this pattern, a large deletion of chromosome 4q12-q32 (>107 Mbp) indicates a strong association with the presence of disseminated tumor cells (DTC) in bone marrow, as well as brain metastases of lung cancer patients [14]. However, the prognostic relevance of this region has not yet been investigated. The aim of this study was to narrow down the relevant hotspot regions and to investigate their prognostic impact in NSCLC. In addition, we sought to analyze whether this loss could also be detected in DTCs of a NSCLC patient.

For this purpose, we first performed allelic imbalance (AI/LOH) analyses in a preliminary study cohort (*n = *86). For further verification, a fluorescence *in situ* hybridization (FISH) analysis of 209 NSCLC patients was performed. A loss within a less than 4 Mb-spanning region at 4q21.23-4q22.1 was identified as a significant negative prognostic marker for disease free as well as overall survival in NSCLC. Furthermore, we performed consecutive immunofluorescence (IF) and FISH analyses on the DTCs of a single NSCLC patient, showing a loss of 4q21.23 in the primary tumor. The same loss could also be detected in the DTCs.

## Materials and Methods

### Samples

This study was approved by the Ethics Committee of the chamber of physicians, Hamburg, Germany. Written informed consent was obtained from all patients. All clinical investigation has been conducted according to the principles expressed in the Declaration of Helsinki. All tumor samples were obtained during surgical resections at the University Medical Centre Hamburg-Eppendorf or associated surgical departments. Clinicopathological data were extracted from a prospective database, and follow-up data were obtained by interviews with the general practitioner or the patient at the outpatient department.

For the allelic imbalance (AI) analyses at 4q, 86 surgically treated primary NSCLC patients with available matched carcinoma and healthy genomic DNA were evaluated for inclusion. The median age of the study cohort was 65.9 years with a predominant male proportion (65.1% versus 34.9%). With regard to lung cancer cell types, 37 patients (43.0%) had a squamous cell carcinoma (SqCC) and 49 (57.0%) an adenocarcinoma (AC). The median follow-up time was 21.4 months (2–60). Further detail is given in S1 Table.

For DNA copy number aberrations (FISH) at 4q, a tissue microarray (TMA), consisting of 209 evaluable primary lung cancer patients, was used, with a median age of 62.3 years at time of surgery. Gender distributions were comparable to the AI study cohort, with a similar predominance of male patients (68.4%). The FISH study cohort encompassed 88 (42.1%) patients with SqCCs, 78 (37.1%) with ACs, 34 (16.3%) with large-cell lung carcinoma, and nine patients (4.5%) with neuroendocrine lung cancer. The median follow up time was 24.7 months (2.5–60). Further details are given in S1 Table.

All patients were reclassified according to the seventh edition of the TNM classification of malignant tumors [15]. In regard to the administration of adjuvant therapy, the following specified criteria have been applied since 2004: ≥ Stage II patients received adjuvant chemotherapy with Cisplatin and Vinorelbin. Staged Ib patients were evaluated for adjuvant therapy if the tumor was >4 cm or in patients with invasion into vein (V+) or invasion into lymphatic vessel (L+). Adjuvant chemotherapy followed by radiation (50–60 Gy) was discussed for ≥ Stage III. Patient characteristics for both study cohorts are shown in S1 Table.

### DNA isolation

Genomic DNA of matched carcinoma (fresh-frozen) and pathologically-verified non-malignant lung tissue or peripheral blood leukocyte, taken prior to surgery was extracted and purified according to the manufacturer's protocol using the QIAamp tissue kit (Qiagen, Hilden, Germany) or InnuPREP DNA Microkit (AnalytikJena, Jena, Germany). If necessary, manual microdissection was performed, in order to obtain a tumor cell content of at least 70%. DNA concentration was determined by NanoDrop ND-1000 Spectrophotometer (Wilmington, DE) and samples were diluted to 10 ng/µl and stored at −20°C until use.

### Allelic imbalance analysis

Based on our previous study, four hotspot regions represented at positions 4q12, 4q21.23, 4q31.2 and 4q35.1 were chosen for further analysis [14]. For each region, two microsatellite markers were used to assess the frequency and clinical relevance of AI (see S2 Table, for details of all microsatellite markers). Forward primers were labeled with a fluorescent dye (6-FAM) for subsequent capillary electrophoresis. PCRs were carried out in a 10 µl reaction mix consisting of 10 ng DNA template, 2.5 mM deoxyribonucleotide triphosphate mix (Invitrogen, Darmstadt, Germany), 2.5 pmol sense and antisense primer (MWG, Ebersberg, Germany), 0.25 U AmpliTaq Gold Polymerase (Applied Biosystems) and 5 µl nuclease-free water. PCR conditions consisted of repeated cycles at 95°C, 60°C−62°C and 72°C for 30 s. For AI determination, capillary electrophoresis with an ABI Prism 3130 Genetic Analyzer (Applied Biosystems, Freiburg, Germany), using a mixture of 40 ml formamide (Hi-Di), 0.2 µl Genescan-500-ROX Standard as well as 0.1 µl of PCR product and denaturation at 94°C for 2 min was performed and the length of allele fragments and fluorescent intensity was assessed. The alleles were defined as the two highest peaks within the expected size range and a ratio of ≥1.5 between the peak heights of the tumor and normal alleles were scored as AI. For overall quality assurance, 10% of used samples were randomly used for repeated analysis. The concordance of allelic status was >99%.

### Fluorescence *in situ* hybridization (FISH) analysis

DNA copy number loss analysis of two hot-spot regions based on the AI analyses was assessed in a larger study population by fluorescence *in situ* hybridization (FISH). Three different BAC probes targeting the regions 2 (RP11-570L13: 4q21.23 and RP11-1053C2: 4q22.1) and 3 (RP11-634D8: 4q31.2) were hybridized on a TMA. The utilized BAC probes were obtained from Source BioScience LifeSciences (Nottingham, UK). One µg of each BAC probe was labeled by random priming (BioPrime Labeling System, Invitrogen) with fluorescently labeled dUTPs (RP11-570L13 and RP11-634D8: Spectrum Orange, RP11-1053C2: Spectrum Green; Enzo Life Sciences, Abbott). As a reference, a centromere probe (CEP 10, SpectrumAqua) was employed (Enzo Life Sciences, Abbott). In order to control the specificity and quality of the fluorescently-labeled BAC probes, the FISH was first tested on metaphase chromosome spreads. Afterwards, protocols for the paraffin slides were established for each BAC probe separately. First, the slides were incubated at 60°C for one hour before they were deparaffinized in xylene and hydrated in an ethanol series. The fixation was achieved by placing the slides into a solution of 2% formaldehyde and methanol at -20°C, followed by rinsing them in phosphate-buffered saline (1x PBS) and pre-treating them with a pre-treatment solution (Invitrogen) at 90°C for 10 min. After PBS washing, the slides were treated with a pre-warmed enzyme-reagent (Invitrogen) at 37°C for 10 min. Again, they were washed in PBS and then dehydrated in an ethanol series. Finally, the slides were denatured at 85°C for 5 min, before they were incubated with probe hybridization mixtures at 37°C overnight. Post-hybridization washing was then performed in 2 x SSC, 0.3% NP-40 buffer at 70°C and room temperature, followed by a second hydration in an ethanol series. DAPI solution was applied for the detection of morphologically intact non-overlapping tumor cells. On average, fluorescent signals of 32 tumor cells for each probe were counted (range 11–57). In order to evaluate the experimental bias, as well as to define the cut-offs of the signal-to-centromere ratio, the hybridization signals of 10 normal control samples (also located on the TMA) were counted. Correspondingly, a ratio >1.5 was defined as a cell carrying a DNA-gain, whereas a ratio <0.75 was defined as a loss.

### Consecutive immunofluorescence staining and FISH analysis

For the isolation of DTCs, mononuclear cells from bone marrow aspirates of NSCLC patients were isolated by FICOLL gradient centrifugation. The cells were then cyto-centrifuged onto glass slides. To detect the DTCs, an immunocytochemistry staining was performed according to the APAAP method (alkaline phosphatase anti alkaline phosphatase), using an antibody against cytokeratins (A45-B/B3, MicroMet, Martinsried, Germany) [16]. An isotype-matched, murine monoclonal antibody (MOPC 21, IgG1; Sigma- Aldrich) served as negative control. DTC positive patients were used for analyzing the loss of 4q22.1 by FISH analysis. FISH was performed on tumor cells that were detected beforehand by IF staining with a Cy3 labeled anti-cytokeratin antibody (A45-Cy3; MicroMet). For cell fixation, solution B (MicroMet) was used. After a PBS washing step, unspecific binding sites were blocked with 10% AB serum (Biotest AG, Dreieich, Germany). Another PBS washing was followed by 45 min A54-Cy3 antibody treatment (1∶300). Cells were again washed and DAPI (CellSearch) was applied for nuclear staining. DTCs were identified in an automated fashion (Ariol Scan, Leica Biosystems, Nussloch, Germany) and localized with the England Finder for re-identification. For consecutive FISH analysis, cells were washed with PBS and incubated for 7 min in a pre-warmed proteinase K (0.1 µg/ml) solution (20 mM Tris-HCl pH 7.5, 0.2% CaCl2xH2O ad 50 ml Aqua dest.). Thereafter, cells were dehydrated in an ethanol series and denatured for 5 min at 75°C (70% Formamide, 0.6 x SSC, pH 7.4). Cells were again dehydrated and probe hybridization mixtures were denatured for 5 min at 75°C. Hybridization was performed over night at 37°C. Post-hybridization washing was done in 50% Formamide/2x SSC, in 2x SSC and in 0.1x SSC at 45°C. After applying DAPI, the DTCs were relocated and fluorescent signals of centromere 3 and RP11-1053C2 probes were counted. A ratio of <0.75 was defined as a loss.

### Statistical analysis

For statistical analysis, SPSS 21.0 for MAC (SPSS Inc., Chicago, IL) was used. Correlation between AI/copy number changes and clinicopathological parameters were assessed by chi-square test. The overall survival (OS) was computed as the period from the date of surgery to either the date of death or last follow-up, whichever occurred first within 60 months. The disease free survival (DFS) was defined as the period from the date of surgery to the date of recurrence or of tumor-related death, whichever occurred first within 60 months. Patients alive without recurrence at the last follow-up date or after 60 months were censored. In-hospital mortality (within 60 days) led to exclusion from survival analysis. Survival was analyzed by using the log-rank test and plotted using the Kaplan–Meier method. If not specified otherwise, results are presented as median survival in months with 95% confidence interval (95% CI). For a multivariate analysis, cox regression hazard model was used to assess the prognostic value of AI/copy number changes. Results are presented as hazard ratio (HR) and 95% CI. Significant statements refer to P-values of two-tailed tests <0.05.

## Results

### Microsatellite analysis

The study cohort used for the microsatellite analysis consisted of a total of 86-matched carcinoma and normal tissue samples of lung cancer patients that underwent surgery. A microsatellite analysis was performed in a total of four regions on chromosome 4q, using two polymorphic markers for each region. Allelic markers close to each other revealed highly similar frequencies of deletion leading to final loss of heterozygosity frequencies of 18.6% (*n = *16) in region 1; 23.3% (*n = *20) in region 2; 25.6% (*n = *22) in region 3 and 34.9% (*n = *30) in region 4. The rate of non-informative cases ranged from 16.3% to 36.0%, depending on the marker. By using two markers for AI detection in each region, the non-informative cases could be reduced to ranges from 2.3% to 15.1%. Regions 1 and 3 had concordant results (normal/loss), whereas region 2 contained three disconcordant results and region 4 contained one of each. All disconcordant results were repeated and the results remained identical, indicating a possible breakpoint between the markers. In total, 45.3% (*n = *39) revealed at least one allelic loss within the analyzed regions. Within these patients, 17.9% (*n = *7) revealed a loss in all regions. Detailed numeric results are given in table 1 and S1 Figure, supporting information.

**Table 1:** Allelotyping results.

	AI	Normal	NI
**region 1**	**16 (18.6)**	**60 (69.8)**	**10 (11.6)**
D4S2978	13 (15.1)	44 (51.2)	24 (27.9)
D4S3000	10 (11.6)	43 (50.0)	31 (36.0)
**region 2**	**20 (23.3)**	**54 (62.8)**	**11 (12.8)**
D4S1534	14 (16.3)	49 (57.0)	17 (19.8)
D4S414	15 (17.4)	44 (51.2)	23 (26.7)
**region 3**	**22 (25.6)**	**50 (58.1)**	**13 (15.1)**
D4S1565	20 (23.3)	48 (55.8)	17 (19.8)
D4S1588	8 (9.3)	30 (34.9)	15 (17.4)
**region 4**	**30 (34.9)**	**54 (62.8)**	**2 (2.3)**
D4S3047	20 (23.3)	47 (54.7)	16 (18.6)
D4S2930	27 (31.4)	43 (50.0)	14 (16.3)

AI: allelic imbalance; NI: non-informative;

values in parenthesis are percentages.

### AI on chromosome 4q and clinicopathological characteristics

There was no evidence of a significant statistical correlation between AI with clinicopathological characteristics in the four investigated regions. However, 83.3% (*n = *15) of patients with AI in region 2 developed a relapse in contrast to 16.7% (*n = *3) without AI (P = 0.073).

### AI on chromosome 4q as a prognostic marker for survival

In univariate survival analyses, a trend towards shorter median disease free (DFS) and overall survival (OS) in patients with AI in region 1 and 2 was evident (median survival difference from 11.9 months to 14 months; details are given in S3 Table). However, differences did not reach statistical significance (P>0.05). Since the study cohort for survival analyses was limited to 68-77 cases, including UICC stage IV patients as well as patients with positive resection margins, confounding effects on survival analysis were to be expected. This was evident for region 2. Therefore, following stratification by age, gender, tumor stage, and resection margins parameters, the presence of AI in region 2 was proven to be an independent prognostic marker for DFS (HR 3.82, *P = *0.044). A similar non-significant trend was also seen for OS (HR 3.56, *P = *0.072). A UICC tumor stage ≥ III was the strongest and only additional negative prognostic marker for DFS and OS, with HR 4.22 (*P = *0.007) and HR 2.87 (P = 0.047) respectively (S4 Table).

### FISH copy number analysis

Based on the reported allelotyping results, we sought to verify these results in a larger study population by FISH analysis. Two different BAC probes, one hybridizing to region 2 and one hybridizing to region 3 were established (S5 Table).

For the region 4q21.23 detected by RP11-570L13, a DNA loss was seen in 24.9% (*n = *52) and a DNA gain in 4.3% (*n = *9) of the analyzed samples. A loss detected at this position revealed a significant histological type-dependent correlation, with 38.0% (*n = *30) loss in SqCC in contrast to 22.7% (*n = *15, *P = *0.049) in AC. The analysis with probe RP11-1053C2 revealed loss in 30.6% and gain in 7.2% (*n = *15), again revealing a significantly more frequent loss rate in SqCC (44.6%, *n = *33) compared to AC (23.3%, *n = *17, *P = *0.022).

Using probe RP11-634D8 (region 3), the rate of successful analysis was 71.6% (*n = *204). A DNA copy number loss was detected in 36.8% (*n = *77) and a gain in 7.7% (*n = *16) of the analyzable samples.

### Copy number loss on chromosome 4q and clinicopathological characteristics

Copy number loss at position 4q21.23 (RP11-570L13) showed a significant correlation with advanced tumor size (*P = *0.005), but with no other pathological characteristics. Copy number loss at position 4q22.1 (RP11-1053C2) revealed a significant association with the presence of lymph node metastases (loss pN0: 25.2%, *n = *26 versus loss ≥pN1: 44.0%, *n = *37; *P = *0.005) as well as advanced UICC stage (loss UICC I: 25.3%, *n = *23 versus loss UICC ≥II: 41.4%, *n = *41; *P = *0.032). Copy number loss at position 4q31.2 (RP11-634D8) showed a significant correlation with advanced tumor size (*P = *0.019). However, no further correlations with clinicopathological characteristics were evident (S5 Table).

### Copy number loss on chromosome 4q as a prognostic marker for survival

**Figure 1:**
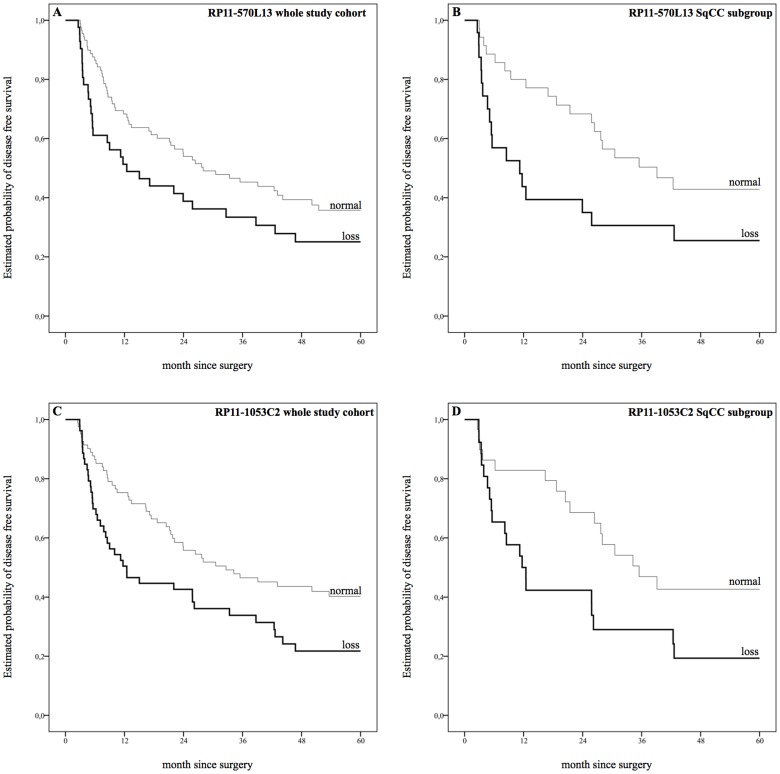
Estimated probability of disease free survival. A: Copy number loss at position 4q21.23 (RP11-570L13) revealed a trend towards shorter median disease free survival (DFS) in univariate analysis (12.5 months versus 28.0 months, *P = *0.056). B: Copy number loss at position 4q21.23 (RP11-570L13) in the squamous cell carcinoma (SqCC) subgroup showed strong correlations with shorter DFS (11.2 months versus 39.1 months) *P = *0.031. C: Copy number loss at position 4q22.1 (RP11-1053C2) showed significant correlations with shorter DFS (12.5 months versus 32.7 months), *P = *0.010. D: Copy number loss at position 4q22.1 (RP11-1053C2) showed significant correlations with shorter DFS in SqCC subgroup (11.7 months versus 35.5 months), *P = *0.027. Survival plots for gain aberrations were faded out for the sake of clarity.

**Figure 2:**
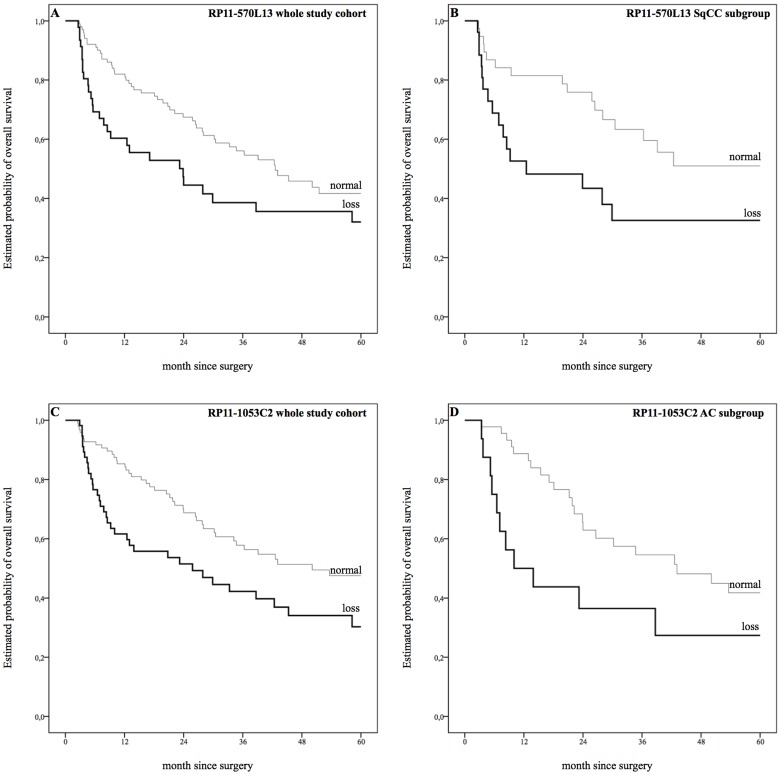
Estimated probability of overall survival. A: Copy number loss at position 4q21.23 (RP11-570L13) revealed strong correlations with shorter median overall survival (OS) in univariate analysis (23.9 months versus 42.6 months), *P = *0.033. B: Copy number loss at position 4q21.23 (RP11-570L13) in the squamous cell carcinoma (SqCC) subgroup showed strong correlations with shorter OS (12.5 months versus 41 months), *P = *0.031 C: Copy number loss at position 4q22.1 (RP11-1053C2) showed significant correlations with shorter DFS (25.8 months versus 40.3 months), *P = *0.012. D: Copy number loss at position 4q22.1 (RP11-1053C2) showed significant correlations with shorter DFS in adenocarcinoma subgroup (10.0 months versus 43.1 months), *P = *0.035. Survival plots for gain aberrations were faded out for the sake of clarity.

In the context of a univariate analysis, copy number loss at position 4q21.23 (RP11-570L13)revealed a non-significant trend towards shorter median DFS (12.5 months versus 28.0 months, *P = *0.056). Subgroup analyses, however, showed a strong correlation with DFS in SqCC subgroup (11.2 months versus 39.1 months, P = 0.031; fig. 1, S6 Table). A similar effect on OS was reported for the whole study cohort (23.9 months versus 42.6 months, *P = *0.033) and SqCC subgroup (12.5 months versus 41 months, *P = *0.031, fig. 2, S6 Table).

Copy number loss at position 4q22.1 (RP11-1053C2) showed significant correlations with shorter median DFS within the whole study cohort as well as within the SqCC subgroup (12.5 months versus 32.7 month, *P = *0.010 and 11.7 months versus 35.5 month, *P = *0.027; fig. 1, S6 Table). Furthermore, a loss of 4q22.1 was associated with shorter OS within the whole study cohort and the AC subgroup, whereas no correlation was seen within the SqCC subgroup (whole study cohort: 25.8 months versus 40.3 months, *P = *0.012; AC subgroup: 10.0 months versus 43.1 months, *P = *0.035, fig. 2, S6 Table).

In contrast, copy number gains did not reveal a correlation with survival at any of the analyzed positions. In line with the results of the AI study cohort, copy number loss at region 3 (RP11-634D8, position 4q31.2) had no impact on survival (S6 Table).

Multivariate analyses identified copy number loss at position 4q21.23 (RP11-570L13) as a negative prognostic marker for DFS and OS for the whole study cohort (cox proportional hazard model, stratified by age, gender, margin clearance, grading and UICC stage; DFS: HR 1.77 (1.10–2.84 95% CI), *P = *0.019; OS: HR 2.20 (1.33–3.64 95% CI), *P = *0.002, table 2) as well as for the SqCC subgroup (DFS: HR 2.85 (1.35–6.04 95% CI), *P = *0.006; OS: HR 2.77 (1.26–6.13 95% CI), *P = *0.012, S7 Table). Similar results were seen for copy number loss at position 4q22.1 concerning the whole study group (RP11-1053C2; DFS: HR 1.64 (1.03–2.61 95% CI), *P = *0.038; OS: 1.84 (1.12–3.01 95% CI), *P = *0.015, table 3), whereas subgroup analysis did not reach statistical significance (S8 Table). A second model of multivariate analyses including the administration of adjuvant chemotherapy was performed (information available in 116 patients). The results stayed significant when the administration of adjuvant chemotherapy was included (data not shown).

**Table 2:** Multivariate analysis RP11-570L13.

	Disease free survival					Overall survival						
	n	(%)	HR	(95% CI)	*P* value	*n*	(%)	HR	(95% CI)	*P* value		
**Age**												
≤62.3	70	(50.4)		reference		77	(49.4)		reference			
>62.3	69	(49.6)	1.03	(0.65–1.62)	0.895	79	(50.6)	1.19	(0.74–1.90)		0.482	
**Gender**												
female	43	(30.9)		reference		50	(32.1)		reference			
male	96	(69.1)	1.03	(0.62–1.71)	0.905	106	(67.9)	1.45	(0.85–2.48)		0.177	
**Margins**												
R0	117	(84.2)		reference		134	(85.9)		reference			
R1	22	(15.8)	1.39	(0.74–2.62)	0.301	22	(14.1)	1.85	(0.99–3.48)		0.056	
**Grading**												
G1/2	77	(55.4)		reference		89	(57.1)		reference			
G3/4	62	(44.6)	1.84	(1.20–2.83)	0.005	67	(42.9)	2.21	(1.40–3.47)		**0.001**	
**UICC stage**												
I	65	(46.8)		reference		73	(46.8)		reference			
II	29	(20.9)	1.85	(1.04–3.27)	**0.035**	37	(23.7)	1.76	(0.96–3.21)		0.067	
III	32	(23.0)	2.94	(1.59–5.45)	**0.001**	33	(21.2)	2.63	(1.39–4.96)		**0.003**	
IV	13	(9.4)	3.52	(1.70–7.27)	**0.001**	13	(8.3)	4.21	(1.95–9.07)		**<0.001**	
**Aberration**												
normal	90	(64.7)		reference		101	(64.7)		reference			
loss	42	(30.2)	1.77	(1.10–2.84)	**0.019**	46	(29.5)	2.20	(1.33–3.64)		**0.002**	
gain	7	(5.0)	1.01	(0.39–2.65)	0.978	9	(5.8)	0.88	(0.33–2.34)		0.801	

Cox regression hazard model was used for multivariate analysis to assess the prognostic value of aberrations.

HR, hazard ratio; CI, confidence interval; UICC, Union for International Cancer Control.

**Table 3:** Multivariate analysis RP11-1053C2.

	Disease free survival					Overall survival				
	*n*	(%)	HR	(95% CI)	*P* value	*n*	(%)	HR	(95% CI)	*P* value
**Age**										
≤62.3	78	(53.1)		reference		85	(51.8)		reference	
>62.3	69	(46.9)	1.15	(0.74–1.79)	0.531	79	(48.2)	1.40	(0.88–2.25)	0.159
**Gender**										
female	45	(30.6)		reference		52	(31.7)		reference	
male	102	(69.4)	1.26	(0.77–2.06)	0.365	112	(68.3)	1.90	(1.10–3.28)	**0.021**
**Margins**										
R0	128	(87.1)		reference		145	(88.4)		reference	
R1	19	(12.9)	1.89	(1.04–3.44)	**0.038**	19	(11.6)	2.16	(1.17–4.00)	**0.014**
**Grading**										
G1/2	84	(57.1)		reference		96	(58.5)		reference	
G3/4	63	(42.9)	1.70	(1.11–2.60)	**0.014**	68	(41.5)	1.83	(1.17–2.85)	**0.008**
**UICC stage**										
I	71	(48.3)		reference		80	(48.8)		reference	
II	33	(22.4)	1.39	(0.78–2.48)	0.266	40	(24.4)	1.29	(0.70–2.37)	0.421
III	29	(19.7)	3.11	(1.75–5.52)	**<0.001**	30	(18.3)	2.61	(1.43–4.76)	**0.002**
IV	14	(9.5)	3.27	(1.73–6.56)	**0.001**	14	(8.5)	3.28	(1.55–6.95)	**0.002**
**Aberration**										
normal	83	(56.5)		reference		97	(59.1)		reference	
loss	53	(36.1)	1.64	(1.03–2.61)	**0.038**	56	(34.1)	1.84	(1.12–3.01)	**0.015**
gain	11	(7.5)	0.71	(0.30–1.68)	0.437	11	(6.7)	0.80	(0.31–2.04)	0.640

Cox regression hazard model was used for multivariate analysis to assess the prognostic value of aberrations.

HR, hazard ratio; CI, confidence interval; UICC, Union for International Cancer Control.

In summary, both positions in region 2, namely 4q21.23 (RP11-570L13) and 4q22.1 (RP11-1053C2), were identified as a negative prognostic marker for DFS as well as OS within the whole study cohort. However, only loss at position 4q21.23 reached significance also in the SqCC subgroup. A flow chart summarizing the survival analyses of the study cohort, the study progress and results are given in S2 and S3 Figures.

### Case report of 4q21.23 loss in DTCs

**Figure 3:**
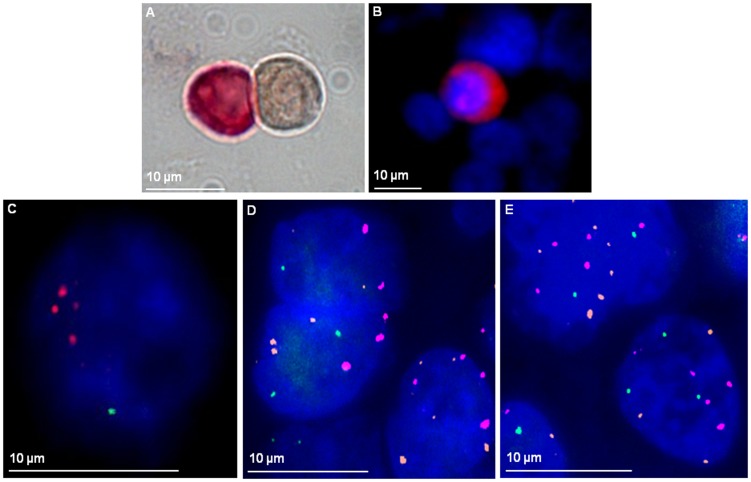
Loss of 4q21.23 in DTCs and tumor tissue of a NSCLC patient. Bone-marrow cells of the patient were immunocytochemically stained against cytokeratin using the APAAP method. A positive DTC (red) and a negative leukocyte (brown) are shown. **B**: Bone-marrow cells of the patient were stained fluorescently against cytokeratin (red signal) followed by FISH analysis in **C**) with RP11-1053C2 probe and Cen3 probe (RP11-1053C2 probe: 1 green signal; Cen3 probe: 3 spectrum orange signals; nuclear staining in DAPI). Cen7 probe (Cen7 probe: spectrum aqua displayed in the pseudo-color magenta) was in addition used for FISH analysis on **D**) primary tumor (2–5 orange signals/cell, 2–4 magenta signals/cell and 0–2 green signals/cell) and **E**) tumor relapse FFPE material (3–6 orange, 4–5 magenta and 1–3 green signals/cell).

Based on our earlier finding that 4q12-32 loss correlates with positive DTC status, we wanted to investigate whether a loss of this region is also present in DTCs from a patient with 4q loss in the primary tumor[14]. A loss of 4q22.1 was detected in 77% (17/22) of the patients DTCs. The range of RP11-1053C2 probe and centromere 3 signals were heterogeneous, varying from 1 to 5 signals (mean 1.7 and 2.7 respectively). FISH analysis was also performed on paraffin-embedded primary tumor and lung relapse material from the same patient (fig. 3). These results again revealed a loss of 4q22.1 and high degree of heterogeneity in the individual cells ranging from 0–4 signals in primary tumor and 0–5 in tumor relapse material.

## Discussion

In a previous study, we were able to identify five chromosomal regions differentiating patients with or without early tumor dissemination. Loss of 4q12-q32 showed the strongest correlation with the positive DTC status. Seventy-three genes within this region were found down regulated among the bone marrow-positive NSCLC patients, indicating a likely presence of tumor suppressor genes within this region [14]. In this study, we were able to narrow down the target region and identify copy number losses within a less than 4 Mb-spanning region on chromosome 4q21.23-22.1 as independent prognostic markers for DFS, as well as OS in NSCLC. The presence of copy number losses within this region was associated with at least 18-month shorter median survival, compared to patients with normal copy numbers. Furthermore, the presence of copy number losses caused an increased risk of disease recurrence and death ranging from 1.6 up to 2.9 within 60 months of follow-up, independent of established prognostic markers for NSCLC (dependent on histological subtype and position of copy number loss). Furthermore, in our case report study, we were able to demonstrate that the loss of chromosomal region 4q21.23 is not only present in both the primary lung tumor and in tumor relapse tissue, but also in a large fraction of very heterogeneous DTCs. This emphasizes the significance of this chromosomal region for tumor dissemination. Uniparental disomy is one mechanism of how a loss of heterozygosity in cells might originate [17]. In NSCLC we are not aware of studies indicating uniparental disomy at 4q. Unfortunately we could not conduct any specific research on whether the AI in our samples was due to uniparental disomy, as the samples for AI and FISH analyses were not overlapping. We were not able to make any statement whether a 4q loss occurs more frequently in a polysomic case of chromosome 4 as we used centromere 10 as reference. In our original paper describing the association between loss of 4q and positive DTC status we investigated 30 NSCLC patients by array CGH. In this rather small number of cases, we could not find any correlation between loss of 4q and total number of chromosomal aberrations [14], indicating that loss of 4q is specifically associated with metastatic behavior, independent of the level of chromosomal instability in the tumor.

Loss of various sized regions at chromosome 4q has been associated with lower survival rates in many types of epithelial cancer including colorectal cancer, ductal pancreatic adenocarcinoma, hepatoblastoma and oral squamous cell carcinoma [3], [18]–[22]. Moreover, in NSCLC, losses of 4q have been associated with metastatic lung adenocarcinomas. However, the size and location of the 4q deletions varies between the different studies [3], [18]–[22].

Besides the identification of the 4q target region in NSCLC and its potential clinical relevance, our data indicates histological subtype-specific deviations in terms of allelic loss frequencies and prognostic relevance. Subgroup analysis revealed a significantly higher rate of allelic loss in SqCC probes in contrast to AC probes. In line with these differences, loss at 4q21.23 (with marker RP11-570L13) within region 2 was identified as a negative prognostic marker for DFS and OS within the whole NSCLC study cohort and especially the SqCC subgroup. This region was not found to be an independent prognostic marker in the AC subgroup. The observed histological subtype-specific allelic loss frequencies with variable influence on survival are in line with former studies reporting several chromosomal regions differentiating SqCC from AC, including the more common loss of 4q in SqCC [5], [8]–[10], [23], [24]. Moreover, numerous studies have indicated that these different genomic alterations of AC and SqCC might provide auspicious opportunities for prospective targeted therapies of lung cancer patients [11]. As the LCLC and neuroendocrine lung cancer subgroups were too small for subgroup analyses, further studies are required in order to assess the potentially deviating impact of allelic imbalances on 4q on these histological subtypes.

Our study cohort did not include precancerous lesions. As a consequence, it is still unclear when these crucial allelic imbalances occur within the timeline of lung cancer pathogenesis. Further studies are needed, in order to evaluate whether the detected 4q hotspot region may serve as a potential diagnostic marker for precancerous lesions or early-stage lung cancer detection, e.g. based on sputum diagnostics as it has been proposed for other genetic aberrations in NSCLC [25].

In conclusion, our data indicates that genomic profiling of NSCLC patients, may be a complementary tool to determine NSCLC patients with aggressive tumor biology and corresponding poor prognosis [26]. Whereas genomic aberrations on the entire chromosome 4q seem to be very frequent, only the loss at 4q21.23-22.1 was shown to be of prognostic relevance in our NSCLC study cohort, especially in SqCC of the lung. Further studies for stratification of the reported results are warranted as well as studies evaluating the presence of potential new tumor suppressor genes within region 4q21.23-22.1.

## Supporting Information

S1 Figure **Allelotyping results.**
(TIFF)

S2 Figure **Flow chart study cohort survival analyses.**
(TIFF)

S3 Figure **Flow chart of the study progress.**
(TIFF)

S1 Table **Cohort characteristics.**
(DOC)

S2 Table **Primers used for allelotyping of each region.**
(DOC)

S3 Table **Univariate survival analysis microsatellite study cohort.**
(DOC)

S4 Table **Multivariate analyses AI study cohort.**
(DOC)

S5 Table **Copy number loss on chromosome 4q and clinicopathological characteristics.**
(DOCX)

S6 Table **Univariate FISH survival analyses.**
(DOC)

S7 Table **Multivariate analyses for 570L13 SqCC subgroup.**
(DOC)

S8 Table **Multivariate subgroup analyses for 1053C2.**
(DOC)
