# Supplementary figures and images for: Loss of 4q21.23-22.1 Is a Prognostic Marker for Disease Free and Overall Survival in Non-Small Cell Lung Cancer
Source: PLoS One. 2014 Dec 11;9(12):e113315. doi: 10.1371/journal.pone.0113315 (PMC4263470; doi:10.1371/journal.pone.0113315)

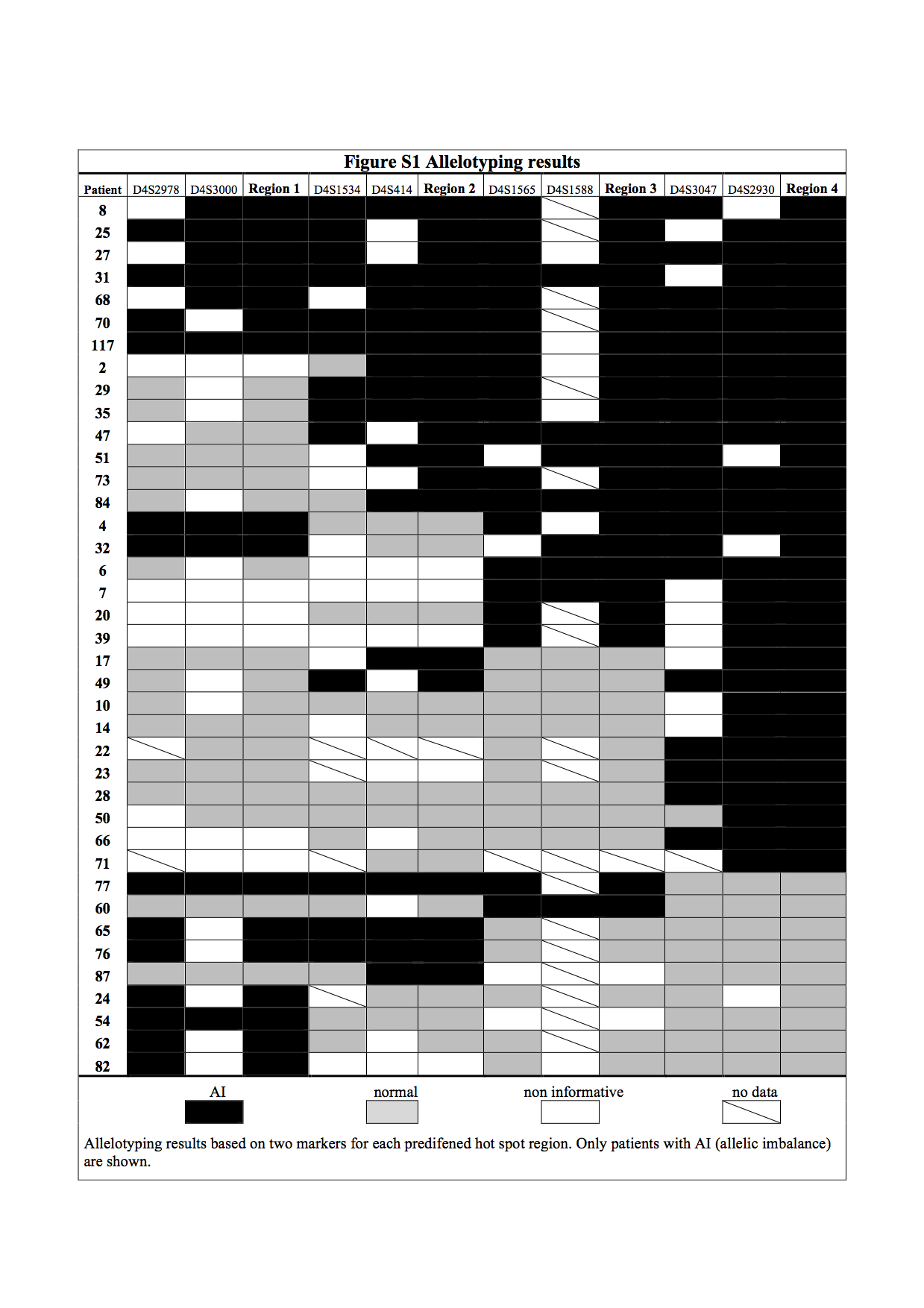

Supplement: S1 Figure — Allelotyping results. (TIFF) [file pone.0113315.s001.tiff]

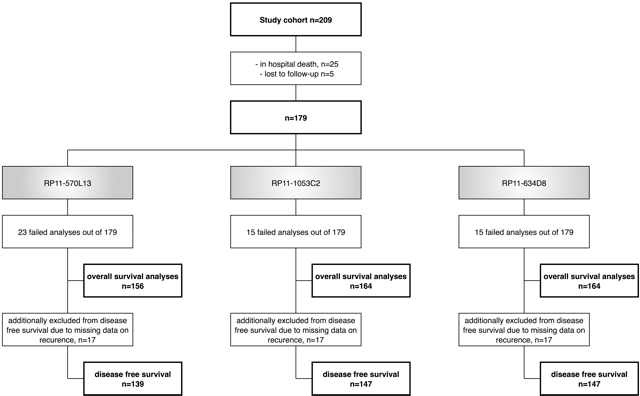

Supplement: S2 Figure — Flow chart study cohort survival analyses. (TIFF) [file pone.0113315.s002.tiff]

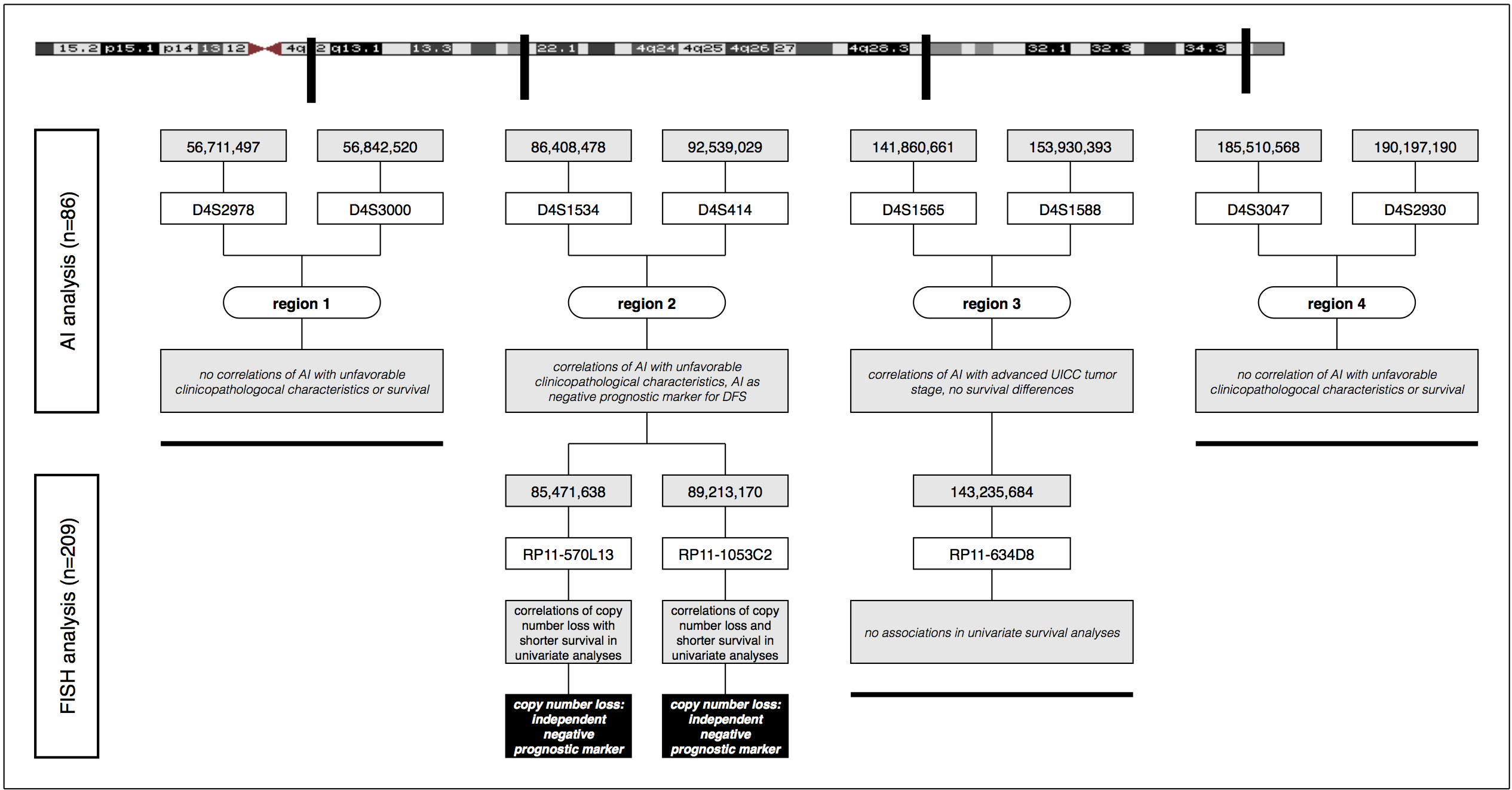

Supplement: S3 Figure — Flow chart of the study progress. (TIFF) [file pone.0113315.s003.tiff]
