# Supplementary material for: Loss of 4q21.23-22.1 Is a Prognostic Marker for Disease Free and Overall Survival in Non-Small Cell Lung Cancer
Source: PLoS One. 2014 Dec 11;9(12):e113315. doi: 10.1371/journal.pone.0113315 (PMC4263470; doi:10.1371/journal.pone.0113315)
Supplement: S1 Table — Cohort characteristics. (DOC) [file pone.0113315.s004.doc]

| **Table S1 Cohort characteristics** | | | | |
| --- | --- | --- | --- | --- |
|  | **AI n=86** | | **FISH n=209** | |
| **Age (yrs.)** |  |  |  |  |
| median (range) | 65.9 (37.5-81.3) | | 62.3 (37.5-91.8) | |
| **Gender** |  |  |  |  |
| female | 30 | (34.9) | 66 | (31.6) |
| male | 56 | (65.1) | 143 | (68.4) |
| **Histological subtype** |  |  |  |  |
| squamous | 37 | (43.0) | 88 | (42.1) |
| adeno | 49 | (57.0) | 78 | (37.1) |
| large cell | 0 | ( 0.0) | 34 | (16.3) |
| neuroendocrine | 0 | ( 0.0) | 9 | ( 4.3) |
| **Neoadjuvant treatment** |  |  |  |  |
| no | 83 | (96.5) | 203 | (97.1) |
| yes | 3 | ( 3.5) | 6 | ( 2.9) |
| **Resection margins** |  |  |  |  |
| R0 | 80 | (93.0) | 177 | (84.7) |
| R1 | 6 | ( 7.0) | 32 | (15.3) |
| **Grading** |  |  |  |  |
| G1/2 | 45 | (52.3) | 123 | (58.9) |
| G3/4 | 41 | (47.7) | 86 | (41.1) |
| **UICC Stage** |  |  |  |  |
| I | 27 | (31.4) | 97 | (46.4) |
| II | 24 | (27.9) | 48 | (23.0) |
| III | 31 | (36.0) | 45 | (21.5) |
| IV | 4 | ( 4.7) | 19 | ( 9.1) |
| **Adjuvant treatment^1,2^** |  |  |  |  |
| no | 49 | (62.0) | 87 | (48.6) |
| yes | 30 | (38.0) | 49 | (27.4) |
| n.a. | 0 | ( 0.0) | 43 | (24.0) |
| **Relapse^1,2,3^** |  |  |  |  |
| no | 25 | (31.6) | 64 | (35.8) |
| yes | 54 | (68.4) | 97 | (54.2) |
| n.a. | 0 | ( 0.0) | 18 | (10.1) |
| **Death^1,2,3^** |  |  |  |  |
| no | 28 | (31.6) | 91 | (50.8) |
| yes | 51 | (64.6) | 88 | (49.2) |
| 1, AI study cohort: exclusion of n=3 due perioperative death and n=4 due to loss to follow up. Final cohort for survival analysis n=79; | | | | |
| 2, FISH study cohort: Exclusion of n=25 due to perioperative death and n=5 due to loss to follow up. Final cohort for overall survival analyses n=179. Final study cohort for disease free survival n=161 due to missing data of n=18; values in parenthesis are percentages; | | | | |
| 3, relapse or death within 60 month of follow up; | | | | |
| FISH: fluorescence in situ hybridization; AI: allelic imbalance; UICC: Union for International Cancer Control. | | | | |
