# Supplementary material for: Loss of 4q21.23-22.1 Is a Prognostic Marker for Disease Free and Overall Survival in Non-Small Cell Lung Cancer
Source: PLoS One. 2014 Dec 11;9(12):e113315. doi: 10.1371/journal.pone.0113315 (PMC4263470; doi:10.1371/journal.pone.0113315)
Supplement: S2 Table — Primers used for allelotyping of each region. (DOC) [file pone.0113315.s005.doc]

| **Table S2 Primers used for allelotyping of each region** | | | | |
| --- | --- | --- | --- | --- |
|  | **Position** | **Forward primer (5´- 3´)** | **Reverse primer (5´- 3´)** | **Annealing temperature** |
| **region 1** |  |  |  |  |
| D4S2978 | 56,711,497 | TGCCAAAGAACTGTGAGC | GTTTTAGGAATAATGGCTGG | 62°C |
| D4S3000 | 56,842,520 | CCTTTGAGTCTCTAGAAAGTCC | CCAGTTTGTATTGGGGTAAC | 62°C |
| **region 2** |  |  |  |  |
| D4S1534 | 86,408,478 | CACCCTGGCTCACTAACATTCTC | TAGACCAGCCCAAGGTAGAGG | 60°C |
| D4S414 | 92,539,029 | TTGCACAAAGCATCAGCCCCTC | TCAGGAACCTCAGCCCATTTAAG | 62°C |
| **region 3** |  |  |  |  |
| D4S1565 | 141,860,661 | ACAAACCCCGATCCTTTTGACT | CCATAAACTGCAGCATCACCA | 62°C |
| D4S1588 | 153,930,393 | CCGGACATCTGAGGTCTTAT | TCCAGAAATGGCTAGAGAGA | 62°C |
| **region 4** |  |  |  |  |
| D4S3047 | 185,510,568 | CCAGAAGGCCCTTGAAGTG | CTCATTTCTGTTTTATTAGGGTG | 60°C |
| D4S2930 | 190,197,190 | CTGTCACCTCACCCCTGTC | GATGATTACTCTTGCATACACTG | 62°C |
|  | | | | |
