# Supplementary material for: Loss of 4q21.23-22.1 Is a Prognostic Marker for Disease Free and Overall Survival in Non-Small Cell Lung Cancer
Source: PLoS One. 2014 Dec 11;9(12):e113315. doi: 10.1371/journal.pone.0113315 (PMC4263470; doi:10.1371/journal.pone.0113315)
Supplement: S3 Table — Univariate survival analysis microsatellite study cohort. (DOC) [file pone.0113315.s006.doc]

| **Table S3 Univariate survival analysis microsatellite study cohort** | | | | | | | | |
| --- | --- | --- | --- | --- | --- | --- | --- | --- |
|  |  |  | **Disease free survival** | | | **Overall survival** | | |
|  | **n** | **(%)** | **median** | **(95% CI)** | ***P* value** | **median** | **(95% CI)** | ***P* value** |
| **Region 1** |  |  |  |  |  |  |  |  |
| Normal | 55 | (79.7) | 24.2 | (16.3-32.2) |  | 29.2 | (13.7-44.7) |  |
| AI | 14 | (20.3) | 10.6 | ( 3.8-17.4) | 0.446 | 16.4 | ( 6.9-25-9) | 0.297 |
| **Region 2** |  |  |  |  |  |  |  |  |
| Normal | 50 | (73.5) | 24.2 | (15.7-32.7) |  | 29.2 | (17.8-40.6) |  |
| AI | 18 | (26.5) | 12.3 | ( 7.3-17.3) | 0.164 | 15.2 | (10.2-20.2) | 0.265 |
| **Region 3** |  |  |  |  |  |  |  |  |
| Normal | 45 | (67.2) | 17.4 | ( 3.0-31.8) |  | 26.6 | (10.6-42.6) |  |
| AI | 22 | (32.8) | 18.8 | ( 5.7-31.9) | 0.979 | 24.4 | (10.8-38.0) | 0.695 |
| **Region 4** |  |  |  |  |  |  |  |  |
| Normal | 48 | (62.3) | 18.8 | ( 2.4-35.2) |  | 23.9 | (12.6-35.2) |  |
| AI | 29 | (37.7) | 22.6 | ( 0.0-49.4) | 0.312 | 26.6 | ( 4.4-48.8) | 0.427 |
| *P* values based on log-rank test; | | | | | | | | |
| median survival time in month; | | | | | | | | |
| CI, confidence interval; AI, allelic imbalance. | | | | | | | | |
