# Supplementary material for: Loss of 4q21.23-22.1 Is a Prognostic Marker for Disease Free and Overall Survival in Non-Small Cell Lung Cancer
Source: PLoS One. 2014 Dec 11;9(12):e113315. doi: 10.1371/journal.pone.0113315 (PMC4263470; doi:10.1371/journal.pone.0113315)
Supplement: S4 Table — Multivariate analyses AI study cohort. (DOC) [file pone.0113315.s007.doc]

| **Table S4 Multivariate analyses AI study cohort** | | | | | | | | |
| --- | --- | --- | --- | --- | --- | --- | --- | --- |
|  | **Disease free survival** | | | | | **Overall survival** | | |
|  | **n** | **(%)** | **HR** | **(95% CI)** | ***P* value** | **HR** | **(95% CI)** | ***P* value** |
| **Age** |  |  |  |  |  |  |  |  |
| ≤66 | 25 | (51.0) |  | reference |  |  | reference |  |
| >66 | 24 | (49.0) | 1.07 | (0.47- 2.47) | 0.872 | 0.76 | (0.33- 1.77) | 0.529 |
| **Gender** |  |  |  |  |  |  |  |  |
| Female | 30 | (61.2) |  | reference |  |  | reference |  |
| Male | 19 | (38.8) | 0.57 | (0.22- 1.42) | 0.228 | 0.99 | (0.39- 2.53) | 0.978 |
| **Margins** |  |  |  |  |  |  |  |  |
| R0 | 46 | (93.9) |  | reference |  |  | reference |  |
| R1 | 3 | ( 6.1) | 1.04 | (0.26- 4.12) | 0.951 | 1.19 | (0.30- 4.75) | 0.810 |
| **UICC stage** | |  |  |  |  |  |  |  |
| I | 17 | (34.7) |  | reference |  |  | reference |  |
| II | 11 | (22.4) | 0.66 | (0.16- 2.75) | 0.568 | 0.54 | (0.12- 2.38) | 0.415 |
| III^1^ | 19 | (38.8) | 4.22 | (1.48-12.02) | 0.007 | 2.87 | (1.01- 8.10) | 0.047 |
| IV^1^ | 2 | ( 4.1) |  |  |  |  |  |  |
| **Region 1** |  |  |  |  |  |  |  |  |
| Normal | 36 | (73.5) |  | reference |  |  | reference |  |
| AI | 13 | (26.5) | 0.87 | (0.32- 2.33) | 0.776 | 0.89 | (0.32- 2.44) | 0.816 |
| **Region 2** |  |  |  |  |  |  |  |  |
| Normal | 33 | (67.3) |  | reference |  |  | reference |  |
| AI | 16 | (32.7) | 3.82 | (1.04-14.13) | 0.044 | 3.56 | (0.89-14.21) | 0.072 |
| **Region 3** |  |  |  |  |  |  |  |  |
| Normal | 32 | (65.3) |  | reference |  |  | reference |  |
| AI | 17 | (34.7) | 1.34 | (0.29- 6.16) | 0.703 | 2.40 | (0.47-12.15) | 0.290 |
| **Region 4** |  |  |  |  |  |  |  |  |
| Normal | 29 | (59.2) |  | reference |  |  | reference |  |
| AI | 20 | (40.8) | 0.46 | (0.11- 1.89) | 0.279 | 0.32 | (0.08- 1.32) | 0.114 |
| Cox regression hazard model was used for multivariate analysis to assess the prognostic value of AI in each region. | | | | | | | | |
| 1, UICC stage III and IV were grouped together in a dominant model. | | | | | | | | |
| HR, hazard ratio; CI, confidence interval; AI, allelic imbalance; UICC,Union for International Cancer Control. | | | | | | | | |
