# Supplementary material for: Loss of 4q21.23-22.1 Is a Prognostic Marker for Disease Free and Overall Survival in Non-Small Cell Lung Cancer
Source: PLoS One. 2014 Dec 11;9(12):e113315. doi: 10.1371/journal.pone.0113315 (PMC4263470; doi:10.1371/journal.pone.0113315)
Supplement: S5 Table — Copy number loss on chromosome 4q and clinicopathological characteristics. (DOCX) [file pone.0113315.s008.docx]

| **Table S5 Copy number loss on chromosome 4q and clinicopathological characteristics** | | | | | | | | | | | | |
| --- | --- | --- | --- | --- | --- | --- | --- | --- | --- | --- | --- | --- |
|  | **570L13^1^** | | |  | **1053C2^2^** | | |  | **634D8^3^** | | |  |
|  | **Normal** | **Loss** | **Gain** | ***P* value** | **Normal** | **Loss** | **Gain** | ***P* value** | **Normal** | **Loss** | **Gain** | ***P* value** |
| **Age** |  |  |  |  |  |  |  |  |  |  |  |  |
| ≤ 62 | 60 (65.9) | 29 (31.9) | 2 ( 2.2) | 0.176 | 55 (57.3) | 37 (57.8) | 4 ( 4.2) | 0.090 | 50 (52.1) | 42 (43.8) | 4 ( 4.2) | 0.095 |
| > 62 | 61 (67.0) | 23 (25.3) | 7 ( 7.7) |  | 56 (59.6) | 27 (28.7) | 11 (11.7) |  | 47 (50.0) | 35 (37.2) | 12 (12.8) |  |
| **Gender** |  |  |  |  |  |  |  |  |  |  |  |  |
| female | 37 (64.9) | 17 (29.8) | 3 ( 5.3) | 0.954 | 35 (61.4) | 18 (28.1) | 4 ( 7.0) | 0.856 | 31 (54.4) | 20 (35.1) | 6 (10.5) | 0.549 |
| male | 84 (67.2) | 35 (28.0) | 6 ( 4.8) |  | 76 (57.1) | 46 (34.6) | 11 ( 8.3) |  | 66 (49.6) | 57 (42.9) | 10 ( 7.5) |  |
| **Histological subtype** |  |  |  |  |  |  |  |  |  |  |  |  |
| squamous | 43 (35.5) | 30 (57.7) | 6 ( 7.6) | 0.056 | 37 (49.3) | 33 (44.0) | 5 ( 6.7) | 0.146 | 31 (41.3) | 39 (52.0) | 5 ( 6.7) | 0.236 |
| adeno | 50 (75.8) | 15 (22.7) | 1 ( 1.5) |  | 53 (68.8) | 17 (22.1) | 7 ( 9.1) |  | 44 (57.1 | 25 (32.5) | 8 (10.4) |  |
| large cell | 23 (79.3) | 4 (13.8) | 2 ( 6.9) |  | 16 (55.2) | 10 (34.5) | 3 (10.3) |  | 16 (55.2) | 10 (34.5) | 3 (10.3) |  |
| neuroendocrine | 5 (62.5) | 3 (37.5) | 0 ( 0.0) |  | 5 (55.6) | 4 (44.4) | 0 ( 0.0) |  | 6 (66.7) | 3 (33.3) | 0 ( 0.0) |  |
| **Tumor size** |  |  |  |  |  |  |  |  |  |  |  |  |
| pT1 | 37 (72.5) | 13 (25.5) | 1 ( 2.0) | 0.005 | 42 (70.0) | 12 (20.0) | 6 (10.0) | 0.069 | 40 (66.7) | 16 (26.7) | 4 ( 6.7) | 0.019 |
| pT2 | 65 (65.7) | 30 (30.3) | 4 ( 4.0) |  | 52 (52.0) | 42 (42.0) | 6 ( 6.0) |  | 43 (43.0) | 50 (50.0) | 7 ( 7.0) |  |
| pT3 | 5 (41.7) | 7 (58.3) | 0 ( 0.0) |  | 5 (38.5) | 6 (46.2) | 2 (15.4) |  | 4 (30.8) | 7 (53.8) | 2 (15.4) |  |
| pT4 | 14 (70.0) | 2 (10.0) | 4 (20.0) |  | 12 (70.6) | 4 (23.5) | 1 ( 5.9) |  | 10 (58.8) | 4 (23.5) | 3 (17.6) |  |
| **Lymphnode status** |  |  |  |  |  |  |  |  |  |  |  |  |
| pN- | 63 (68.5) | 26 (28.3) | 3 ( 3.3) | 0.474 | 71 (68.9) | 26 (25.2) | 6 ( 5.8) | 0.005 | 59 (57.3) | 37 (35.9) | 7 ( 6.8) | 0.100 |
| pN+ | 54 (62.8) | 26 (30.2) | 6 ( 7.0) |  | 38 (45.2) | 37 (44.0) | 9 (10.7) |  | 35 (41.7) | 40 (47.6) | 9 (10.7) |  |
| **Grading** |  |  |  |  |  |  |  |  |  |  |  |  |
| G1/2 | 68 (65.4) | 33 (31.7) | 3 ( 2.9) | 0.226 | 70 (61.9) | 36 (31.9) | 7 ( 6.2) | 0.389 | 57 (50.4) | 48 (42.5) | 8 ( 7.1) | 0.645 |
| G3/4 | 53 (67.9) | 19 (24.4) | 6 ( 7.7) |  | 41 (53.2) | 28 (36.4) | 8 (10.4) |  | 40 (51.9) | 29 (37.7) | 8 (10.4) |  |
| **UICC Stage** |  |  |  |  |  |  |  |  |  |  |  |  |
| I | 57 (68.7) | 24 (28.9) | 2 ( 2.4) | 0.079 | 62 (68.1) | 23 (25.3) | 6 ( 6.6) | 0.081 | 53 (58.2) | 32 (35.2) | 6 ( 6.6) | 0.420 |
| II | 28 (70.0) | 11 (27.5) | 1 ( 2.5) |  | 20 (45.5) | 22 (50.0) | 2 ( 4.5) |  | 18 (40.9) | 22 (50.0) | 4 ( 9.1) |  |
| III | 23 (54.8) | 13 (31.0) | 6 (14.3) |  | 19 (50.0) | 14 (36.8) | 5 (13.2) |  | 16 (42.1) | 17 (44.7) | 5 (13.2) |  |
| IV | 13 (76.5) | 4 (23.5) | 0 ( 0.0) |  | 10 (58.8) | 5 (29.4) | 2 (11.8) |  | 10 (58.8) | 6 (35.3) | 1 ( 5.9) |  |
| **Relapse** |  |  |  |  |  |  |  |  |  |  |  |  |
| no | 37 (74.0) | 12 (24.0) | 1 ( 2.0) | 0.177 | 37 (66.1) | 14 (25.0) | 5 ( 8.9) | 0.091 | 31 (55.4) | 22 (39.3) | 3 (5.4) | 0.440 |
| yes | 53 (59.6) | 30 (33.7) | 6 ( 6.7) |  | 46 (50.5) | 39 (42.9) | 6 ( 6.6) |  | 42 (46.2) | 40 (44.0) | 9 (9.9) |  |
| **Death** |  |  |  |  |  |  |  |  |  |  |  |  |
| no | 53 (70.7) | 18 (24.0) | 4 ( 5.3) | 0.316 | 55 (66.3) | 22 (26.5) | 6 ( 7.2) | 0.112 | 45 (54.2) | 32 (38.6) | 6 (7.2) | 0.735 |
| yes | 48 (59.3) | 28 (34.6) | 5 ( 6.2) |  | 42 (51.9) | 34 (42.0) | 5 ( 6.2) |  | 39 (48.1) | 35 (43.2) | 7 (8.6) |  |
| ^1^ failed FISH analysis in 27 out of 209 patients, correlation with tumor relapse based on 139 patients, correlation with death based on 156 patients. See also figure S2: flow chart of survival analyses | | | | | | | | | | | | |
| ^2,3^ failed FISH analysis in 19 out of 209 patients, correlation with tumor relapse based on 147 patients, correlation with death based on 164 patients. See also figure S2: flow chart of survival analyses | | | | | | | | | | | | |
| ^1,2,3^ missing data on lymph node status in 4 patients | | | | | | | | | | | | |
