# Supplementary material for: Loss of 4q21.23-22.1 Is a Prognostic Marker for Disease Free and Overall Survival in Non-Small Cell Lung Cancer
Source: PLoS One. 2014 Dec 11;9(12):e113315. doi: 10.1371/journal.pone.0113315 (PMC4263470; doi:10.1371/journal.pone.0113315)
Supplement: S6 Table — Univariate FISH survival analyses. (DOC) [file pone.0113315.s009.doc]

| **Table S6 Univariate FISH survival analyses** | | | | | | | | | | | | | | | |
| --- | --- | --- | --- | --- | --- | --- | --- | --- | --- | --- | --- | --- | --- | --- | --- |
|  | **All** | | | | | **Squamous cell carcinoma** | | | | | **Adenocarcinoma** | | | | |
|  | **n** | **(%)** | **month** | **(95% CI)** | ***P* value** | **n** | **(%)** | **month** | **(95% CI)** | ***P* value** | **n** | **(%)** | **month** | **(95% CI)** | ***P* value** |
| **Marker and aberration** | | | **Disease free survival** | | | | | | | | | | | | |
| **570L13** |  |  |  |  |  |  | |  |  |  |  | |  |  |  |
| normal | 90 | (64.7) | 28.0 | (13.8-42.3) |  | 36 | (55.4) | 39.1 | (21.1-57.2) |  | 36 | (73.5) | 11.8 | ( 7.5-16.1) |  |
| loss | 42 | (30.2) | 12.5 | ( 2.3-22.6) | **0.056** | 24 | (36.9) | 11.2 | ( 1.7-20.7) | **0.031** | 12 | (24.5) | 17.1 | ( 5.2-29.0) | 0.778 |
| gain | 7 | ( 5.0) | 20.5 | ( 0.0-48.6) | 0.116 | 5 | ( 7.7) | 20.5 | ( 0.0-44.0) | 0.116 | 1 | ( 2.0) | 38.3 | ( 0.0-48.8) | 0.859 |
| **1053C2** |  |  |  |  |  |  | |  |  |  |  | |  |  |  |
| normal | 83 | (56.5) | 32.7 | (15.2-50.1) |  | 31 | (50.8) | 35.5 | (21.9-49.1) |  | 38 | (65.5) | 21.8 | (13.8-29.8) |  |
| loss | 53 | (36.1) | 12.5 | ( 5.4-19.5) | **0.010** | 26 | (42.6) | 11.7 | ( 6.7-16.7) | **0.027** | 15 | (25.9) | 7.8 | ( 3.3-12.2) | 0.081 |
| gain | 11 | ( 7.5) | 38.3 | (22.3-54.3) | 0.965 | 4 | ( 6.6) | 29.6 | (13.6-45-7)^§^ | 0.998 | 5 | ( 8.6) | 38.3 | ( 0.0-95.2) | 0.669 |
| **634D8** |  |  |  |  |  |  | |  |  |  |  | |  |  |  |
| normal | 73 | (64.7) | 30.5 | (14.1-47.0) |  | 27 | (44.3) | 30.5 | (16.4-44.7) |  | 30 | (51.7) | 21.8 | (13.3-30.3) |  |
| loss | 62 | (29.5) | 22.0 | ( 6.9-37.2) | 0.325 | 30 | (49.2) | 25.8 | ( 6.8-44.8) | 0.574 | 22 | (37.9) | 10.0 | ( 2.4-17.6) | 0.617 |
| gain | 12 | ( 5.8) | 22.5 | ( 2.7-42.3) | 0.367 | 4 | ( 6.6) | 20.5 | ( 4.4-36.6) | 0.615 | 6 | (10.3) | 11.8 | ( 0.0-48.8) | 0.506 |
| **Marker and aberration** | | | **Overall survival** | | | | | | | | | | | | |
| **570L13** |  |  |  |  |  |  | |  |  |  |  | |  |  |  |
| normal | 101 | (64.7) | 42.6 | (28.8-56.4) |  | 38 | (54.3) | 41.0 | (33.7-48.2)^§^ |  | 42 | (68.9) | 26.6 | (11.9-41.3) |  |
| loss | 46 | (29.5) | 23.9 | ( 8.8-39.0) | **0.033** | 26 | (37.1) | 12.5 | ( 0.0-35.4) | **0.031** | 14 | (23.0) | 24.0 | (13.1-34.9) | 0.911 |
| gain | 9 | ( 5.8) | 26.6 | (14.5-38.8) | 0.298 | 6 | ( 8.6) | 26.6 | (17.2-36.0) | 0.292 | 1 | ( 1.6) | 38.3 | (17.3-80.4) | 0.884 |
| **1053C2** |  |  |  |  |  |  | |  |  |  |  | |  |  |  |
| normal | 97 | (59.1) | 40.3 | (35.7-44.8)^§^ |  | 33 | (50.8) | 38.6 | (30.5-46.8)^§^ |  | 46 | (68.7) | 43.1 | (17.3-68.9) |  |
| loss | 56 | (34.1) | 25.8 | ( 6.4-45.2) | **0.012** | 28 | (43.1) | 27.9 | (15.6-40.2) | 0.263 | 16 | (23.9) | 10.0 | ( 0.0-21.0) | **0.035** |
| gain | 11 | ( 6.7) | 38.3 | (26.7-49.9)^§^ | 0.823 | 4 | ( 6.2) | 38.9 | (29.1-48.7)^§^ | 0.563 | 5 | ( 7.5) | 38.3 | ( 0.0-80.4) | 0.334 |
| **634D8** |  |  |  |  |  |  | |  |  |  |  | |  |  |  |
| normal | 84 | (51.2) | 43.1 | (27.3-58.9) |  | 28 | (43.1) | 36.3 | (20.9-51.7) |  | 36 | (53.7) | 42.6 | (22.4-62.8) |  |
| loss | 67 | (40.9) | 34.2 | (16.8-51.7) | 0.352 | 33 | (50.8) | 34.2 | (14.6-53.8) | 0.862 | 24 | (35.8) | 15.4 | ( 0.0-46.3) | 0.387 |
| gain | 13 | ( 7.9) | 33.3 | (16.9-49.7) | 0.489 | 4 | ( 6.2) | 26.6 | (22.5-50.1) | 0.994 | 7 | (10.4) | 38.3 | (17.3-59.3) | 0.582 |
| *P* values based on log-rank test; CI, confidence interval; DFS, disease free survival; OS, overall survival;§, mean, median not reached. | | | | | | | | | | | | | | | |
