# Supplementary material for: Loss of 4q21.23-22.1 Is a Prognostic Marker for Disease Free and Overall Survival in Non-Small Cell Lung Cancer
Source: PLoS One. 2014 Dec 11;9(12):e113315. doi: 10.1371/journal.pone.0113315 (PMC4263470; doi:10.1371/journal.pone.0113315)
Supplement: S7 Table — Multivariate analyses for 570L13 SqCC subgroup. (DOC) [file pone.0113315.s010.doc]

| **Table S7 Multivariate analyses for 570L13 SqCC subgroup** | | | | | | | | | | |
| --- | --- | --- | --- | --- | --- | --- | --- | --- | --- | --- |
|  | **Disease free survival** | | | | | **Overall survival** | | | | |
|  | **n** | **(%)** | **HR** | **(95% CI)** | ***P* value** | **n** | **(%)** | **HR** | **(95% CI)** | ***P* value** |
| **Age** |  |  |  |  |  |  | |  |  |  |
| ≤62.3 | 29 | (44.6) |  | reference |  | 29 | (41.4) |  | reference |  |
| >62.3 | 36 | (55.4) | 0.92 | (0.43-1.98) | 0.919 | 41 | (58.6) | 1.22 | (0.54-2.80) | 0.633 |
| **Gender** |  |  |  |  |  |  | |  |  |  |
| female | 18 | (27.7) |  | reference |  | 19 | (27.1) |  | reference |  |
| male | 47 | (72.3) | 1.91 | (0.30-2.04) | 0.156 | 51 | (72.9) | 2.20 | (0.85-5.70) | 0.104 |
| **Margins** |  |  |  |  |  |  | |  |  |  |
| R0 | 53 | (81.5) |  | reference |  | 58 | (82.9) |  | reference |  |
| R1 | 12 | (18.5) | 0.79 | (0.30-2.04) | 0.622 | 12 | (17.1) | 1.40 | (0.53-3.70) | 0.493 |
| **Grading** |  |  |  |  |  |  | |  |  |  |
| G1/2 | 50 | (76.9) |  | reference |  | 55 | (78.6) |  | reference |  |
| G3/4 | 15 | (23.1) | 3.19 | (1.45-6.99) | **0.004** | 15 | (21.4) | 3.82 | (1.63-9.00) | **0.002** |
| **UICC stage** |  |  |  |  |  |  |  |  |  |  |
| I | 30 | (46.2) |  | reference |  | 32 | (45.7) |  | reference |  |
| II | 16 | (24.6) | 2.88 | (1.18-6.99) | **0.020** | 19 | (27.1) | 2.34 | (0.89-6-14) | 0.085 |
| III^1^ | 16 | (24.6) | 5.10 | (2.08-12.52) | **<0.001** | 19 | (22.9) | 5.18 | (1.95-13.74) | **0.001** |
| IV^1^ | 3 | (4.6) |  |  |  | 3 | ( 4.3) |  |  |  |
| **Aberration** |  |  |  |  |  |  |  |  |  |  |
| normal | 36 | (55.4) |  | reference |  | 38 | (54.3) |  | reference |  |
| loss | 24 | (36.9) | 2.85 | (1.35-6.04) | **0.006** | 26 | (37.1) | 2.77 | (1.26-6.13) | **0.012** |
| gain | 5 | ( 7.7) | 1.18 | (0.32-4.35) | 0.808 | 6 | ( 8.6) | 0.65 | (0.16-2.68) | 0.548 |
| Cox regression hazard model was used for multivariate analysis to assess the prognostic value of aberrations. | | | | | | | | | | |
| 1, UICC stage III and IV were grouped together in a dominant model. | | | | | | | | | | |
| HR, hazard ratio; CI, confidence interval; UICC,Union for International Cancer Control. | | | | | | | | | | |
