# Supplementary material for: Loss of 4q21.23-22.1 Is a Prognostic Marker for Disease Free and Overall Survival in Non-Small Cell Lung Cancer
Source: PLoS One. 2014 Dec 11;9(12):e113315. doi: 10.1371/journal.pone.0113315 (PMC4263470; doi:10.1371/journal.pone.0113315)
Supplement: S8 Table — Multivariate subgroup analyses for 1053C2. (DOC) [file pone.0113315.s011.doc]

| **Table S8 Multivariate subgroup analyses for 1053C2** | | | | | | | | | | |
| --- | --- | --- | --- | --- | --- | --- | --- | --- | --- | --- |
|  | **Disease free survival** | | | | | **Overall Survival** | | | | |
|  | **Squamous cell carinoma** | | | | | **Adenocarcinoma** | | | | |
|  | **n** | **(%)** | **HR** | **(95% CI)** | ***P* value** | **n** | **(%)** | **HR** | **(95% CI)** | ***P* value** |
| **Age** |  |  |  |  |  |  | |  |  |  |
| ≤62.3 | 28 | (45.9) |  | reference |  | 37 | (56.9) |  | reference |  |
| >62.3 | 33 | (54.1) | 0,81 | (0.37-1.78) | 0.594 | 30 | (46.2) | 0.91 | (0.44-1.91) | 0.809 |
| **Gender** |  |  |  |  |  |  | |  |  |  |
| female | 14 | (23.0) |  | reference |  | 23 | (35.4) |  | reference |  |
| male | 47 | (77.0) | 1.84 | (0.64-5.26) | 0.259 | 44 | (67.7) | 3.33 | (1.43-7.74) | **0.005** |
| **Margins** |  |  |  |  |  |  | |  |  |  |
| R0 | 51 | (83.6) |  | reference |  | 61 | (93.8) |  | reference |  |
| R1 | 10 | (16.4) | 0.99 | (0.40-2.45) | 0.992 | 6 | ( 9.2) | 3.13 | (1.17-8.42) | **0.024** |
| **Grading** |  |  |  |  |  |  | |  |  |  |
| G1/2 | 47 | (77.0) |  | reference |  | 38 | (58.5) |  | reference |  |
| G3/4 | 14 | (23.0) | 2.72 | (1.17-6.32) | **0.020** | 29 | (44.6) | 2.87 | (1.27-6-51) | **0.012** |
| **UICC stage** |  |  |  |  |  |  |  |  |  |  |
| I | 26 | (42.6) |  | reference |  | 29 | (44.6) |  | reference |  |
| II | 18 | (29.5) | 1.49 | (0.60-3.72) | 0.392 | 13 | (20.0) | 1.38 | (0.49-3-89) | 0.544 |
| III* | 14 | (23.0) | 4.88 | (1.92-12.40) | **0.001** | 14 | (21.5) | 1.78 | (0.68-4.65) | 0.240 |
| IV* | 3 | (4.9) |  |  |  | 11 | (16.9) | 1.65 | (0.59-4.66) | 0.342 |
| **Aberration** |  |  |  |  |  |  |  |  |  |  |
| normal | 33 | (54.1) |  | reference |  | 46 | (70.8) |  | reference |  |
| loss | 28 | (45.9) | 2.04 | (0.96-4.32) | 0.062 | 14 | (21.5) | 2.00 | (0.90-4.48) | 0.091 |
| gain | 4 | ( 6.6) | 0.63 | (0.13-2.99) | 0.557 | 5 | ( 7.7) | 1.80 | (0.57-5-67) | 0.313 |
| Cox regression hazard model was used for multivariate analysis to assess the prognostic value of aberrations. | | | | | | | | | | |
| 1, UICC stage III and IV were grouped together in a dominant model. | | | | | | | | | | |
| HR, hazard ratio; CI, confidence interval; UICC,Union for International Cancer Control. | | | | | | | | | | |
